# Pathology and Treatment of Ovarian Diseases

**Published:** 1875-02

**Authors:** Lawson Tait


### PATHOLOGY AND TREATMENT OF OVARIAN DISEASES.

*Abstract from the Hastings Prize Essay by Lawson Tait, F. R. C S.*

The remaining affections of the ovary are those which are the result of increased growth, usually taking the form of cystic degeneration. More rarely the growth is solid, and may be either fibromyxomatous, or, more commonly, cancer. There are no diseases in the province of surgery where more care is needed in weighing every point in the history, every symptom and sign, for the purpose of establishing an accurate diagnosis, than in those classed under the head of ovarian tumors. It is best to make, first of all, a mental list of all the conditions that might exist, and exclude one after another until the alternative is left.

From the history alone, no ovarian tumor can be diagnosticated. The rate of increase gives no guide. The details given by the patient as to the region in which the growth was first observed are often very misleading. Tumors of one ovary are often stated by the bearers to have originated in the side opposite to that from which they are found to grow. The menstrual histories are to be almost disregarded in making the diagnosis. With some menorrhagia, with others amenorrhœa, may occur. It is especially important to eliminate pregnancy, particularly the condition of hydramnois, which the author has known to be treated with fatal results, on two occasions, by tapping. The uterus, in the early months of normal pregnancy, is not unfrequently displaced to one or other side, and has often been mistaken for an ovarian cyst.

For the diagnosis of ovarian tumors, there are various and almost numberless symptoms, the great majority being of little or no consequence for accuracy, and none of them are trustworthy. In the early growth of a simple cyst, symptoms of any kind are seldom met with until the tumor is sufficiently large to be impacted within the pelvis. The growth of dermoid cysts, on the contrary, is often accompanied by intense pain. As a rule, pain is not met with until cystic tumors are large enough, if out of the pelvis, to press on important viscera, or unless the surface undergoes inflammatory change. As it enlarges, the symptoms become more varied and numerous. In the pelvis, its pressure gives rise to dysuria or incontinence, constipation or diarrhœa, and to various neuralgias; in the abdominal cavity, by pressure on the stomach, liver and diaphragm, it often produces nausea and vomiting, distaste for food, etc. Coincidently, there appear indications of great systemic alterations.

Ordinarily, the pressure of an ovarian tumor is not brought to the surgeon's notice till it has reached a sufficient size to rise out of the pelvis and appear as an abdominal enlargement. Sometimes, however, it is necessary to determine the nature of a small pelvic tumor. An ovarian tumor, in this case, will be found to be almost invariably behind the uterus. Usually, this organ can be fixed between the two hands; behind it is the tumor, and if the uterus can be moved independently of it, and if the tumor can also be raised out of the pelvis, no doubt need be felt that it is a tumor of the ovary or of the broad ligament; how to determine between these two it is hard to say, nor is it of much consequence.

As the tumor increases in size and rises out of the pelvis, it becomes more difficult to determine that it is not intimately associated with the uterus. It is often necessary to introduce the sound to determine this point; but, as a rule, this ought never to be done at the first examination. It not unfrequently happens that menstruation goes on for a few months after conception, and to assert the diagnosis between an early pregnancy and an ovarian tumor just rising out of the pelvis, at a first examination, is a task which only the rash or the greatly experienced will undertake. Only when it has been ascertained, by manipulation, that the uterus is not enlarged, may the sound be introduced. If then it be ascertained that the tumor is not uterine, that it is rounded, elastic and capable to some extent, of being raised out of the pelvis, it is almost certainly ovarian. It may be ovarian if fixed, though it is rarely adherent at so early a stage of growth. If fixed, it may be a hæmatocele, an abscess, or a soft tumor growing from bone; previous history, symptoms, and, above all, exploration by the aspirator, will determine these points.

When an ovarian tumor has risen out of the pelvis, and has met with none of the accidents to which it is liable, its diagnosis is easy. Palpation and percussion will eliminate phantom tumors. Fluctuation will assist in determining whether it be unilocular or multilocular. Two conditions must be carefully excluded: cystic disease of the uterus and hydramnios. In the former, the tumor will be found associated with the uterus, the latter moving along with it when moved, and being dragged upwards by it to an extent that ought always to make us cautious.

Solid uterine tumors, besides the absence of fluctuation, have in addition two vascular signs not met with in ovarian growths, namely: An aortic impulse, which may be seen and felt, and an enlargement of the uterine arteries, to be felt in the vagina.

Hydramnios generally occurs in twins. Ballotement will assist in determining the different diagnosis between a unilocular ovarian cyst and distended uterus.

If the tumor be found to be not uterine and solid, yet attached to the uterus, and moving it so as to lead to the belief that it is ovarian, we have a choice between a dermoid cyst, a fibrous tumor of the ovary, cancer of the ovary, or a pedunculated fibrous tumor of the uterus. Fluctuation in some part, and its peculiar nodulated character, will betray the dermoid cyst, while fibrous tumors of the ovary and cancer are very rare.

The main difficulties in the diagnosis of an ovarian tumor are met with in the subsequent stages of its growth, between the time when it has risen above the brim of the pelvis, as far as the umbilicus, until it has reached its extremest size. Fluctuation, of so much use at an earlier stage, comes to have a decreasing value. Percussion will generally show, in an ovarian tumor, the characteristic distribution of dullness, though accidental complications may vitiate the value of this sign.

The *tactus eruditus* of a practiced ovariotomist can recognize—when both an ovarian tumor and ascites are present at the same time—that there is a double wave of fluctuation; one superficial and rapid, due to the ascitic fluid, and another deeper and perceptibly less rapid, due to the fluid in the cyst.

The enlargement of veins often seen in the skin of the abdomen in cases of ovarian tumor is of no great assistance as a diagnostic sign. Auscultation gives chiefly negative signs. Tapping, either for the removal of ascitic fluid or the contents of a cyst, is often a great help towards an accurate diagnosis. By the removal of peritoneal dropsy, we may discover the actual relations of an ovarian tumor, or we may find that the supposed tumor has no existence, and by removing the contents of a unilocular tumor, or of one or more of the major cysts of a multilocular growth, we may determine the existence of pelvic adhesions, of pregnancy, or of some other condition that may alter our views as to treatment.

Formerly, great stress was laid on the diagnosis of adhesions, but modern experience has led to a disregard, almost wholly, of adhesions that are not visceral or pelvic.

A final means for purposes of diagnosis, a *dernier resort* in cases of doubt, is the exploratory incision. The experience gained by the operator from one such case ought to assist him in avoiding its necessity in similar doubtful cases.

Mr. Spencer Wells has characterized the condition of the medical treatment of ovarian tumors as one of hopeless impotence.

The surgical treatment of ovarian tumors has now been simplified into two operations: the minor operation of tapping, which is palliative, and rarely curative, and the major operation of ovariotomy, which is either curative or fatal. Tapping by the vagina is not usually attended with good results.

The proper selection of cases for the performance of ovariotomy is one of difficulty, and can be based on experience alone. In the author's opinion there can only be two reasons for refusing to do ovariotomy—either that the case is not far enough advanced, or that the tumor, in all probability, could not be removed. The most unfavorable case for ovariotomy is to be found in a young, healthy woman, with a medium-sized tumor. The rule ought to be to delay an ovariotomy as long as is consistent with the patient's chances of recovery, bearing in mind that it is not the healthiest that recover best.

Presupposing that a proper case has been selected, experience shows that the more nearly the patient's surroundings resemble those of a healthy private house the better. She requires some preparations for the change that is about to be made in her alvine actions. The time of the operation should be about midway between two menstrual periods. As to the anæsthetic to be employed, the author objects to chloroform, on account of the vomiting which follows its use, and he thinks sulphuric ether is not much better. He recommends the bichloride of methylene and the methylene ether. (The writer then goes on to state his method of operating.)

If there be no adhesions, and no large secondary cysts, ovariotomy, thus far, is a very simple operation. The complications and unsuspected difficulties are endless, and tax the presence of mind and ingenuity of the operator. Thus, a second dermoid cyst may be found packed down in the pelvis, and it may be very difficult to remove it. For securing the pedicle, Mr. Wells's calliper-clamp is preferred.

Any tumor of the uterus had better be let alone, unless it be markedly pedunculated. If the uterus be enlarged by pregnaucy, it must not be interfered with; but if unfortunately punctured in mistake for a cyst, it is best to lay it open and empty it.

The after-course of a case of ovariotomy is subject to many mishaps. Of their approach, the temperature curve is the most trustworthy indication. Immediately after the operation, the temperature almost invariably falls considerably. To obviate the shock, it is well to place hot-water bottles to the sides and feet, and administer a diffusible stimulant. Advantage has resulted from the practice of giving a subcutaneous injection of morphia immediately after the operation.

For the first twenty-four hours after ovariotomy, the patient is allowed no other sustenance than ice or iced water, and, perhaps, in case of sickness, a little soda-water and brandy, or champagne. Nutriment may be given cautiously on the second day, in the form of chicken-broth or beef-tea, in small quantities, frequently, so as to obviate vomiting. No solid food to be taken till after the fourth day.

In the event of the occurrence of symptoms of peritonitis, special interference may be necessary, such as opening the recto-uterine *cul-de-sac* from the vagina for drainage. Septic poisoning is no more a peculiarity of ovariotomy than it is of amputations.

Vomiting, a frequent and troublesome symptom, must be stopped, if possible. The most useful remedy in Mr. Tait's experience is Morson's pepsin wine, given in drachm doses every ten minutes with a little ice-water.

Flatulence is often a distressing symptom, and, if accompanied by a high temperature, is pathognomonic of peritonitis. Milk and lime-water often mitigate it, and the passage of a Burns's tube, as far as possible, up the rectum, will give much relief. Failing that, the author has frequently punctured the distended bowels with a fine exploring trocar, and kept it in for some hours, with great relief. Inflammatory attacks of the chest and diarrhœa sometimes occur. For three or four days after the operation, the catheter should be used every six or seven hours. The bowels should be kept closed by opium for seven or eight days. After the wound has healed, the patient should wear a tight-fitting abdominal belt instead of stays; for, in spite of all care in inserting stitches, there is a proneness to the formation of ventral hernia in the cicatrix for many months after the newness of the union has passed off.

The pathology of ovarian cysts involves a number of questions that have been raised and discussed by observers of the greatest eminence, but thus far there are no very satisfactory explanations of the growths. As to the causes of ovarian dropsy, we must confess that we know nothing about them. The most common form, the adenoid or proliferous, and also the rare multiple tumors, occur during the period of life when ovarian cell-growth is mature; the more rare unilocular cystic growths, besides being met with during this period, occur at the extremes of life.

The author has not yet met an ovarian tumor that was unilocular, and he believes that all unilocular tumors in the neighborhood of the ovary are not ovarian, but of parovarian origin. The parovarian consists of a few closed linear sacs, the remains of the tubules of the Wolffian body in fœtal life, which may readily be seen on holding the broad ligament with the ovary and Fallopian tube *in situ*, up to the light. These tubules frequently contain a perceptible amount of fluid, and are frequently accidentally found in *post mortem* examinations, distended to the size of beans or filbert nuts. In every truly unilocular tumor, Mr. Tait has found the ovary unaffected, though on several occasions he has seen it stretched over the cyst-wall.

Mr. Tait has met with an example of a rare variety of ovarian tumors, the origin of which has been traced to Rokitansky and Ritchie. In the case recorded by the author, both ovaries were affected in their entirety. The tumors were multilocular, and had one or two major, with innumerable minor cysts, graduating down to the most minute size. The tumors had the appearance of huge white raspberries. An examination of the contents of a large number of the cysts discovered in every one more or less distinct remains of an ovum. The condition seemed to be an hypertrophy of the ovaries, with arrested development of their contents.—*Boston Medical and Surgical Journal.*

